# Genome editing and assisted reproduction: curing embryos, society or prospective parents?

**DOI:** 10.1007/s11019-017-9793-y

**Published:** 2017-07-19

**Authors:** Giulia Cavaliere

**Affiliations:** Wellcome Trust PhD Student in Bioethics & Society, Department of Global Health & Social Medicine, King’s College London, London, UK

**Keywords:** Genome editing, Assisted reproduction, Genetic kinship, PGD, Therapy, Selection

## Abstract

This paper explores the ethics of introducing genome-editing technologies as a new reproductive option. In particular, it focuses on whether genome editing can be considered a morally valuable alternative to preimplantation genetic diagnosis (PGD). Two arguments against the use of genome editing in reproduction are analysed, namely safety concerns and germline modification. These arguments are then contrasted with arguments in favour of genome editing, in particular with the argument of the child’s welfare and the argument of parental reproductive autonomy. In addition to these two arguments, genome editing could be considered as a worthy alternative to PGD as it may not be subjected to some of the moral critiques moved against this technology. Even if these arguments offer sound reasons in favour of introducing genome editing as a new reproductive option, I conclude that these benefits should be balanced against other considerations. More specifically, I maintain that concerns regarding the equality of access to assisted reproduction and the allocation of scarce resources should be addressed prior to the adoption of genome editing as a new reproductive option.

## Introduction: genetic diseases, genome editing and existing alternatives

Different reproductive options are available for couples or individuals at risk of transmitting genetic diseases to their offspring who wish to have children. In this paper, I explore ethical and social questions raised by the use of genome editing into the context of assisted reproduction and, in particular, as a potential alternative to preimplantation genetic diagnosis (PGD).

Some of the reproductive options available to this group of individuals include refraining from having genetically related children and/or using technologies to reduce or avoid the risk of transmission. The first set of options includes adopting existing children or turning to third-party reproduction (i.e. relying on a gamete donor). Adoption is currently legal in many European countries, but eligibility criteria vary. For instance, in some countries, access to this practice is limited to married heterosexual couples (e.g. Italy), while other countries have wider access criteria and allow same-sex couples (e.g. the Netherlands and the United Kingdom) and single parents (e.g. France and the United Kingdom) to adopt. In addition, other criteria such as marital status and age play a role in the decision to grant adoption.

Another possibility to avoid transmission of genetic diseases is for individuals to have partly genetically-related children and to seek gamete donors. This is commonly referred to as third-party reproduction, which allows couples to have children who are genetically related to a donor and to the unaffected individual in the couple. Third-party reproduction is currently only legal in some countries (e.g. the United Kingdom, the Netherlands and Spain) and usually restricted to heterosexual couples. Moreover, the state only subsidises IVF with donor gametes in a few countries (Gianaroli et al. 2016).

Alternatively, prospective parents at risk of transmitting genetic conditions to their offspring can seek to procreate with the aid of assisted reproductive technologies (ARTs) and preimplantation screening technologies (such as PGD), which would allow them to have genetically related children free from the condition that affects them (or one of them). PGD allows the testing of embryos created with IVF for genetic abnormalities prior to their transfer in utero. This technology is currently legal in many European countries (Gianaroli et al. 2016), but in some countries it remains restricted to so-called ‘serious’ conditions (e.g. in Italy and Germany), and in others is completely banned (e.g. in Poland and Switzerland; Biondi 2013; Gianaroli et al. 2016). Across Europe, eligibility criteria vary. In the United Kingdom, for instance, the Human Fertilisation and Embryology Authority (HFEA) periodically revises and updates the lists of conditions that are eligible for screening with PGD. Other countries, such as Germany and Italy, recently approved the use of PGD, but access to this practice remains restricted to a very limited number of severe, early onset conditions (Biondi 2013; Gianaroli et al. 2016).

### PGD and assisted reproduction

Where PGD is legal, it is typically used in cases where both prospective parents are carriers of an autosomal recessive mutation. These mutations are responsible for the occurrence of autosomal recessive monogenic diseases (i.e. diseases caused by a mutation in a single gene) such as cystic fibrosis and sickle cells anaemia.^1^ When both prospective parents are carriers of such mutations, future offspring have a 1 in 4 chance of inheriting the mutated gene and developing an autosomal recessive disease, while they have a 1 in 2 chance of inheriting one abnormal gene and thus becoming healthy carriers. PGD allows the testing and selection of embryos created through IVF to transfer in utero those that are either free from the abnormal gene related to the prospective parents’ condition (or that are carriers of such mutated gene when no mutation-free embryo is obtained). PGD is also effective in cases where one of the prospective parents is heterozygous for an autosomal dominant mutation, meaning that they carry two different variants of a gene. Autosomal dominant mutations are responsible for the occurrence of diseases such as Huntington’s and neurofibromatosis type 1. Future offspring have a 1 in 2 chance of developing autosomal dominant diseases even if only one of the prospective parents is affected, because it is possible that the embryo would carry the ‘good’ genetic variant from both parents. If the embryo inherited the disease-causing variant from only one parent, however, the resulting child would be affected by the disease.

It could be the case that none of the embryos created through IVF is free from the undesirable genetic mutation. For instance, when one of the prospective parents is homozygous for a dominant genetic disorder, the risk of transmission to offspring is as high as 100%, and hence no mutation-free embryos can be obtained. In addition, when prospective parents are both heterozygous for a dominant genetic disorder, the risk of transmission is as high as 75%, hence the chances of finding mutation-free embryos significantly low. Another case where PGD is not effective is when both parents are homozygous for a recessive genetic disorder, meaning that they both carry two variants of the disease-causing gene (Nuffield Council on Bioethics 2016; Vassena et al. 2016). In such cases, genome editing could represent an alternative to PGD and a new reproductive option for some prospective parents: mutations potentially leading to monogenic diseases would be corrected in embryos created with IVF prior to the transfer in utero or directly onto prospective parents’ gametes prior to fertilisation. Lastly, gene editing could replace PGD for women at risk of transmitting mitochondrial diseases as mitochondrial DNA mutations present in oocytes^2^ could be corrected in the embryo (Vassena et al. 2016).

In the following section, I briefly present the debate on genome editing technologies applied to human embryos and I show how these technologies could be used as an alternative to PGD for the aforementioned cases where PGD is not effective. In “Assisted reproduction and PGD, or assisted reproduction and CRISPR?” section, I present the moral reasons in favour of and against introducing genome editing as an alternative to PGD. In particular, I present arguments in favour of using genome editing instead of, or as an alternative to, PGD, and argue that some of the moral arguments against PGD would not be applicable to genome editing. I conclude, ad interim, that such arguments offer a *prima facie* case in favour of introducing genome editing as a new reproductive option, given that safety concerns are thoroughly assessed. In “Curing embryos, society or prospective parents?” section, I turn to other arguments on the ethics of introducing genome editing as a new reproductive option and argue that there are additional questions that need to be carefully addressed. I conclude that introducing genome editing in the context of assisted reproduction would have some benefits, but that concerns regarding the equality of access to assisted reproduction and the allocation of scarce resources should be addressed beforehand.

## CRISPR and assisted reproduction

Gene-editing technologies have been around for over a decade. Zinc finger nucleases (ZFNs) and transcription activator-like effector nucleases (TALENs), two gene-editing technologies, were discovered in 2005 and 2010 respectively (Nuffield Council on Bioethics 2016). ZENs and TALENs are relatively precise techniques, but have the disadvantage that they need engineered proteins to target specific sequences of the DNA, a procedure that requires time and resources (Nuffield Council on Bioethics 2016).

A new gene editing technique sparked debate early in 2015 due to its application on non-viable human embryos by a group of Chinese scientists (Baltimore et al. 2015; Lanphier and Urnov 2015). The technique in question is CRISPR/Cas9, an RNA-guided tool composed of two parts: clustered regularly interspaced short palindromic repeat (CRISPR) and CRISPR-associated protein 9 (Cas9). CRISPR/Cas9 makes use of a naturally occurring defence mechanism that bacteria use to avoid harmful infections caused by pathogenic organisms (e.g. viruses). The RNA tool (CRISPR) functions as a guide for the Cas proteins to target specific parts of the genome, which are subsequently cut by the Cas proteins. These cut strands can be exploited to modify the nucleotide sequence of DNA and to insert genes at the cut site. The application of this technique to human embryos and human gametes (i.e. oocytes and sperm cells) has been widely criticised for a number of issues, but chiefly for its potential to introduce *inheritable changes* in the human genome (germline modification). Indeed, the issue of germline modification has catalysed the attention of many scientists and ethicists (Brokowski et al. 2015; Lander 2015; Lanphier and Urnov 2015).

This paper focuses on PGD and CRISPR^3^ applications to the field of assisted reproduction. In particular, it focuses on CRISPR as a potential alternative to PGD. CRISPR could represent a tool to avoid the occurrence of genetic diseases in future children through the modification of the genetic makeup of embryos created with IVF from couples with a known risk of transmitting such genetic diseases. Since using CRISPR on early embryos could give to prospective parents who are either affected by monogenic diseases or who are carriers of them a chance to avoid the transmission of these diseases to their offspring, this particular application of CRISPR can be considered a new reproductive option for parents who want to have genetically related children.

## Assisted reproduction and PGD, or assisted reproduction and CRISPR?

Research on human embryos with CRISPR technology is still at an early stage and only few experiments have been carried out thus far (Vassena et al. 2016). Despite this, the issue of allowing clinical research has been discussed recently (Gyngell et al. 2016; Vassena et al. 2016; Reyes and Lanner 2017). The two main precautionary reasons that have been advanced against clinical applications of genome editing on human embryos or gamete cells are concerns regarding introducing changes in the human germline and safety questions. Many scholars and members of the public consider germline modifications unethical and a “line that should not be crossed” (Collins 2015; for a discussion of this claim, see: Camporesi and Cavaliere 2016). The worry is that edited embryos will pass their edited genome on to future generations, thus introducing changes in humanity’s gene pool. While it is of fundamental moral importance to consider the impact of present actions that could potentially have an impact on future generations, it seems reductive to limit these precautionary reflections to changes introduced with genome editing technologies on reproductive cells and embryos. In particular, those who worry about germline modifications via CRISPR and other genome editing technologies maintain that there is something exceptional in changes introduced *technologically* in our genomes via genome editing (and indirectly into the genomes of our offspring). The worry about germline modification encompasses a number of concerns, including the view that the human genome should be preserved intact as a “common heritage of our humanity” (cf. UNESCO statement against cloning, UNESCO 1997); the view that would be ethically problematic to change the germline of future generations “without their consent” (Collins 2015); and concerns regarding the safety of the technique not only for the child born thanks to its aid, but also for the child’s children (more about this below and in “Reproductive autonomy, child welfare and the interests of society” section). This first view misrepresents partially the natural history of humankind and how past and present humanly introduced innovations shape future generations (Buchanan 2011; Harris 1992). The introduction of agriculture, for instance, played a role not only in shaping our environment, but has fundamentally changed our genomes. The same could be said about technologies such as literacy and numeracy, which laid the foundations for technological innovations that have significantly changed us (Buchanan 2008, 2011). In other words, from a moral point of view, it seems irrelevant which *means* are used and whether inheritable changes are introduced with genome editing technologies or caused by other technological innovations, unless one is able to show the moral exceptionality of using genome editing technologies (Harris 2010). In addition to this, focusing solely on technical means to introduce changes the human gene pool overlooks how other policies (such as those dealing with greenhouse gas mitigation), innovations (such as those in the field of agriculture) and human habits could have similar effects (i.e. introduce changes in the gene pool) with potentially much more serious consequences (Dupras et al. 2014). The view that emphasises the need to ask the consent of future generations, as argued by Harris (2016), fails to state how such consent could be obtained. Most procreative decisions affect future generations, but it is unclear how and why the consent of future offspring should be obtained prior to act (Harris 2016).

The other argument against allowing genome editing for clinical uses is concern for the safety of future offspring (and of this offspring’s offspring). At this stage, safety is indeed an issue and the efficiency of genome editing on embryos remains low, with mosaic embryos (i.e. embryos that have abnormal numbers of chromosomes in certain cells resulting in genetically different cells coexisting in the same organism) being the main known drawback of these technologies (Vassena et al. 2016). Despite this, some studies have proven the feasibility of gene editing in animals (Heo et al. 2014; Shao et al. 2014; Yoshimi et al. 2014; Zou et al. 2015), even though the efficiency of genetically modifying zygotes with Cas9 ranges between 0.5 and 40% (Araki and Ishii 2014). In addition, a recent study demonstrated the feasibility of preventing the onset of a genetic disorder such as cataract development (Wu et al. 2013) and the injection of Cas9 into primate zygotes led to the birth of genetically modified offspring (Liu et al. 2014; Niu et al. 2014).

### The case for genome editing: two sets of arguments

There are two sets of arguments for introducing CRISPR and other gene editing technologies into the clinic, provided that safety concerns are properly addressed. In this section I first outline the first group of arguments, which concerns the benefits of genome editing for future children (and their children too) and for prospective parents (Gyngell et al. 2016; Reyes and Lanner 2017). In the following section, I present additional reasons why genome editing could be a morally preferable alternative to PGD: genome editing would not be subjected to some of the critiques moved against PGD.

The moral reasons that ground the case for PGD (the welfare of future children and the reproductive autonomy of prospective parents. Pennings et al. 2007; Buchanan et al. 2001; Harris 1992) can be extended to defend the clinical use of genome editing in reproduction. It is widely accepted that reproductive autonomy and respect for parental discretion in reproduction are values worth defending^4^ (Buchanan et al. 2001; Harris 1992; Robertson 1996). With respect to reproductive autonomy, genome editing would be comparatively better than PGD: it would offer an alternative to this technology for those aforementioned cases where PGD is not effective or for prospective parents who wish to increase their chances of having mutation-free embryos. In this sense, genome editing could be said to enhance reproductive autonomy. With respect to the welfare of the child, the case in favour of genome editing seems *prima facie* stronger than the case in favour of PGD. Unlike the latter technology, whereby embryos implanted can be carriers of the parents’ mutated gene, genome editing would allow modification of the genetic makeup of embryos who would consequently develop into mutation-free offspring. In other words, genome editing would prevent the occurrence of genetic diseases in future generations, while PGD can sometimes only prevent the occurrence of genetic diseases in the child that develops from the implanted embryo (Gyngell et al. 2016).

There are, however, other arguments in favour of preferring genome editing to PGD. PGD is a contested practice as its scopes are not therapeutic (i.e. PGD does not *treat* embryos) but rather selective (i.e. PGD selects the embryos that should be transferred in utero. Asch and Barlevy 2012; Parens and Asch 2003). PGD as a means to select embryos that have a decreased risk of developing into a child with a genetic condition is seen as ethically troubling for two reasons: firstly, because it goes against the traditional ends of medicine and ‘selects out’ rather than ‘cures’ persons affected by genetic conditions (MacKellar and Bechtel 2014), and secondly, because decisions on which embryos should be selected are said to embody value judgements regarding people living with certain disabilities (Knoppers et al. 2006; Parens and Ash 2003), a critique of screening technologies that became known as the ‘expressivist argument’ or ‘expressivist objection’ (Buchanan 1996; Shakespeare 2006).

### Selection versus therapy

PGD (at the moment) and CRISPR (potentially in the future) are two technologies that enable similar ends: in both cases, these technologies increase the chances of giving parents genetically related offspring unaffected by specific genetic conditions. Despite the similarity of the outcomes (i.e. healthy child), the means used are rather different. PGD is a form of genetic testing that allows screening for abnormalities in early embryos and to subsequently implant only those with a decreased risk of developing a certain condition. Instead, CRISPR and other gene editing technologies are tools for gene therapy that allow the modification of embryos or of gamete cells in order to avoid the occurrence of certain conditions in the future child (and in future generations).

Following this distinction of means, there is a sense that while PGD entails the *selection* of embryos, CRISPR is more akin to *therapy*. At this point, however, it is important to note that CRISPR and other genome editing technologies can be considered both therapeutic and non-strictly-therapeutic (or, following Wrigley et al. “pre-emptively therapeutic”; Wrigley et al. 2015, p. 636). I am not trying to violate Aristotle’s principle of non-contradiction on the impossibility that contradictory assertions can be both true at the same time here. What I mean is rather that whether these technologies are therapeutic depends on what sort of factual and moral considerations are taken into account. If the focus is on the prospective parents, then CRISPR can be considered therapeutic in some instances because it could be a solution (or a treatment?) for those couples who would not otherwise be able to conceive children that are related to them and that are free from the risk of developing (or have a decreased risk to develop) the condition that affects them.

If the focus is on the future children, we have two possible interpretations: following the view that equates embryos with persons, CRISPR *is* therapeutic because it treats the embryos (i.e. it treats persons), whereas PGD is selective because it selects in/out the embryos (i.e. it selects out persons). If, however, we are more inclined to think of embryos as beings with the *potential* to develop into persons (i.e. potentiality view, arguably a more widely shared position), then CRISPR is not straightforwardly therapeutic, because there is no person to be treated at the moment that we use the technology.^5^ Despite this remark, I argue that there is a sense whereby genome editing can still be considered therapeutic, or, as mentioned above, pre-emptively therapeutic. In order to assess whether CRISPR can be considered pre-emptively therapeutic, it is necessary to determine whether embryo X (i.e. the embryo that exist prior to the application of CRISPR) is identical to new-born X^+ about 9 months^ (i.e. the child that is born after the application of CRISPR on embryo X). This assessment matters for the ethical debate on PGD and genome editing because if these two entities (embryo X and new-born X^+ about 9 months^) are identical, *then* PGD would be more problematic than CRISPR as the first would be a selective technology, whereas the second would be a therapeutic technology. A brief explanation of the question of identity is needed before proceeding with the discussion on PGD and CRISPR and the ethics thereof. Currently, ethicists and philosophers involved in the debate on reproductive genetic technologies seem to be divided on whether genome editing technologies applied to embryos are identity-affecting technologies or not, as this largely depends on the circumstances taken into account.^6^ When I say “identity-affecting” I refer to the idea of numerical identity and to the metaphysical problem of determining how we can rightly refer to one and the same person in any different set of circumstances, despite the changes that the person undergoes over time. Thus, for instance, there is numerical identity between a person X and a person Y only if person X and Y are the same person. To put it simply, I am numerically identical to the person that is writing this paper at the moment. The challenge of any account of numerical identity is then to explain what determines the entity that we in fact are despite the changes that we undergo over time. In this sense, if I grow taller or if I lose an eye due to an accident, I am still numerically identical to the entity I was before having that accident or when I was shorter. This is the case because changes such as losing an eye or growing taller are largely considered *contingent* to numerical identity, namely they do not change the entity that I am.

Returning to genome editing, those who do not subscribe to the embryos as persons view can view the technology in two different ways. The contentious matter is whether applying CRISPR on embryo X creates a numerically different entity (call it embryo Z, that will eventually develop into person Z) or it just leads to a numerically identical entity (call it embryo X*, that will eventually develop into person X*) in the same sense that applying gene therapy on adult X does not create a different adult Z, but only leads to a numerically identical adult X*. While in the first case genome editing would be considered an identity-affecting technology (i.e. a technology that by virtue of its use creates an entirely new entity), in the second case it would amount to a non-identity-affecting technology.^7^ Following the first interpretation, CRISPR cannot be considered a therapy as, by virtue of its use on an embryo, it determines the kind of person that is brought into being rather than pre-emptively curing the same pre-person. On the contrary, if we are inclined to follow the second interpretation, then CRISPR is therapeutic as it pre-emptively cures an embryo that will develop into a numerically identical child that does not have the genetic condition that is consciously avoided.^8^ It is only in this second sense that it is possible to say that if the genome of an embryo affected by a certain genetic condition is modified and this condition eradicated, then this embryo will develop into a numerically identical child who, had CRISPR not been used, would have been affected by a genetic disease. As a consequence, even if one does not subscribe to the embryo-as-persons view, *there is a sense* whereby genome editing can be considered at least *more similar* to therapy than to selection: genome editing would be a pre-emptive treatment for the genetic disease that is caused by the genetic mutation at the embryonic stage.

If the second interpretation about genome editing being non-identity-affecting is embraced, then both the teleological objection (i.e. PGD is morally problematic because it does not fall within the traditional ends of medicine) and the selective attitudes objection (i.e. PGD is morally problematic because it promotes selective and discriminatory attitudes) seem to be less applicable to the use of genome editing on embryos to prevent the occurrence of certain conditions in future children. As explained above, editing the genome of embryos can be considered pre-emptively therapeutic and thus falls within (or at least closer to) the traditional ends of medicine. From this, it also follows that it would be problematic to consider such practice as selective or discriminatory: disability scholars would have to condemn all the interventions aimed at treating genetic diseases (Barnes 2014).

These clarifications have normative implications, namely that, once the safety of editing the genome of human embryos is carefully assessed, the latter technology should be considered preferable to PGD. In the next section, I will outline some additional questions that need to be addressed and explain why preferring CRISPR over PGD is not completely cost-free.

## Curing embryos, society or prospective parents?

In the previous sections, two main questions have remained unaddressed. One question is on the value and meaning of genetic parenthood. Another, albeit related, question concerns the ethics of existing alternatives. I explore these two questions in this last section and conclude that they provide at least some *prima facie* moral reasons for carefully considering the introduction of a new reproductive option when similar options are already available.

A peculiar feature of assisted reproductive technologies such as PGD, and possibly genome editing, is that they are often offered to prospective parents who are affected by a genetic condition in order to conceive (or increase their chances of conceiving) healthy offspring. It is in this sense that these technologies represent a *solution* for those prospective parents whose *problem* is the impossibility of having a *genetically related* and *healthy* child; or at least healthier than the child that would otherwise be brought into the world had these technologies not be employed. As explained in the first section of this paper, there are other options than PGD to increase the chances of having healthy children, but they entail refraining from having genetically related children (for one individual in the couple or, in the case of adoption, both parties). Reproductive technologies such as PGD and genome editing convey the interests of different groups: the prospective parents, the future offspring and the society where these offspring will grow and thrive. Despite the importance of all three stakeholders, their interests are not granted equal importance: the welfare of future children and the reproductive autonomy of the prospective parents are usually considered of greater moral importance than the aggregate interests of society in having healthy members, respecting competing values on assisted reproduction, and limiting the use of certain technologies against a backdrop of scarce resources. This is what I define as the received view on the ethics of assisted reproductive technologies. An ethical assessment of whether introducing new technologies in the context of reproduction should thus consider these three aspects (with the aforementioned prioritisation in mind) in turn.

### Reproductive autonomy, child welfare and the interests of society

Genome editing, at first sight, seems to score high on the reproductive autonomy and welfare of the child fronts: unlike PGD, it allows for more conditions to be corrected and the reduction of the occurrence of certain genetic conditions in future generations; it also increases the reproductive autonomy of the parents by offering not only one more possibility in the geneticists tool-box, but also by allowing those couples for whom PGD is not always successful to have biologically related, healthy offspring. So far so good. Or maybe not? The idea that more choice leads to greater freedom has been challenged (Dworkin 1982; Rose 1999; Rothman 1985). More options can also translate into more uncertainties, and greater perceived and actual responsibilities for the prospective parents (Dworkin 1982). In this sense, introducing genome editing into the clinic as an alternative to PGD may be detrimental for the very same prospective parents that it is designed for. While genome editing may be more routinely employed in the future, some issues will likely remain. These issues include, for instance, reflections upon which conditions should be eligible for the use of genome editing and whether parents who fail to employ the most efficient technology available could be considered morally responsible (Rothman 1985).

What about the welfare of the future child? The empirical question of whether safety concerns will be put to rest and genome editing will ever be *safe enough* to represent a concrete alternative to PGD divides scholars (Harris 2016). The reasons for this are twofold: first, no one knows the answer to such questions *yet*. Secondly, this empirical question is strongly influenced by the value judgements of scientists, ethicists, policy-makers and the public on the degree of certainty required to move forward. Hence, even without denying that such empirical questions will be eventually be put to rest, it is still important to note that a consensus on the question of safety will be hard to reach due to the competing values at stake in stakeholders’ assessments. Those taking a precautionary stance concerning technological development will favour existing technologies over the newly discovered, while those who are generally in favour of technological development will be ready to accept a higher degree of risk in the name of such progress and of the potential benefits that it may yield. With respect to the safety and the welfare of the future child, whether genome editing really represents a better option than PGD will thus divide scholars, scientists and the public (and, as exemplified by the debate on embryo-applications of CRISPR, already does). A decision on whether to allow genome editing will thus have to rest not only on a thorough assessment of the safety of the techniques, but also on a democratic process that takes into account such differing views and values (Cavaliere 2017; Jasanoff et al. 2015; Kitcher 2001). The ethical assessment of new techniques ought to not only rest on a cost/benefit analysis, but also on an evaluation of existing alternatives, including those that do not rely on biomedical means. In other words, whether genome editing really represents a worthy alternative to existing options (such as PGD) depends on the extent to which the welfare of the future child can be put at risk to allow couples to have a genetically related child. Regulators and ethicists that argue in favour of eventually replacing PGD with genome editing, and couples for whom PGD does not represent an option, will have to consider whether reproductive autonomy should trump questions on the welfare of the child in light of uncertainty.

Lastly, what role should societal interests and views play in the decision over whether genome editing should replace PGD? There are different ways in which assisted reproductive technologies and procreative decisions more generally impinge on society. Procreative decisions influence the *type* and the *number* of people that will be created. They allow new consumers, producers, workers, mothers, fathers, etc. to come into existence. We live in an increasingly interlinked world and the aggregate effects of individual decisions affect a wider range of people than ever before (Singer 2004). There are historical reasons why third parties’ interventions in procreation are looked at with suspicion, and the shadow of eugenics seems to extend over any discussion regarding reproductive technologies and their governance (Paul 1992). Despite these worries, the regulation of new reproductive technologies will be influenced by governments’ policies, which in turn will reflect the interests of society and societal views on emerging reproductive technologies. Regarding the governance of genome editing technologies and their potential use in the context of assisted reproduction, the interests of society might play a role in two main ways: the first is whether genome editing is ethically acceptable for a large segment of society (Kitcher 2001), and second, related, is whether existing alternatives warrant the introduction of a new practice and the clinical research necessary to safely implement it. Almost every new technology introduced or discussed for potential introduction in reproduction seems to stir controversies. The recent debates on genome editing (Camporesi and Cavaliere 2016), mitochondrial replacement techniques (Appleby 2015) and ‘older’ debates on PGD (Scott 2006) are just a few instances of these controversies. However, once certain uses are constrained and lines drawn (for instance between therapeutic and enhancing uses), these technologies have been approved and, at least in certain countries, accepted by large swaths of the population. Thus, even if genome editing will be met with controversies and will encounter resistance, it does not *prima facie* translate into the need for banning any research involving it. On the contrary, this should translate into support for a democratic and deliberative approach to the governance of technological innovation (Jasanoff et al. 2015) and into the respecting of competing moral views on these issues (Cavaliere 2017).

### Societal interests and the costs of introducing genome editing in the context of assisted reproduction

At this point, there is, however, there is one last thing to consider, which concerns the aforementioned interests of society and how they should and could play a role in the ethical assessment of introducing genome editing in the context of assisted reproduction. While it is true that genome editing could open up new reproductive possibilities for certain couples (i.e. enhance reproductive autonomy) and provide heritable benefits to their future offspring (i.e. considerations regarding the welfare of future child), these benefits ought to be balanced against the costs of introducing a new reproductive technology. These costs include the investment of public resources, considering both the scarcity of such resources and the existence of available alternatives. Emanuel et al. (2000) argue that for clinical research to be ethical, among other requirements, it needs to have social value, namely it should be directed at “a diagnostic and therapeutic intervention that could lead to improvements in health and well-being” (Emanuel et al. 2000). Being of social value is an ethical requirement for clinical research to go forward precisely because it operates in a context of scarce resources. From this it follows that if the social value of a technology is limited, then the investment of public resources for the development and implementation of such technology may be unethical (Rulli 2016b). The proposed clinical research (in this case that needed in order to implement genome editing as an alternative to PGD) needs to be evaluated on two levels: absolute and relative. The absolute level is settled once the proposed research is expected to bring about improvements to health and well-being. The relative level, however, needs more: the proposed research (and the improvements to health and well-being thereof) needs to be compared both with other potential uses of those scarce resources and with existing alternatives to bring about similar improvements to health and well-being. Two of the criteria that are often employed to assess whether to invest resources in certain clinical research and whether it will bring about significant improvements to health and well-being are the severity of the condition and the number of individuals that it affects (Rulli 2016b). If we consider these two criteria, the benefits of the introduction of genome editing as a new reproductive option are arguably minor and thus may not warrant the investment of public resources. The number of cases for which PGD is not an option, as mentioned in the first section, is limited. In addition, considering the importance of taking into account future children’s welfare, the unresolved questions concerning safety seem to indicate that health improvements may not be so significant. An obvious critique to this is the following: clinical research is aimed at improving techniques in order to achieve significant benefits for future children. This is certainly correct and we would not enjoy the benefits of many technologies and drugs if it was not for clinical research. But again: resources are limited and not all research can be publicly funded.

Returning to the relative level to evaluate clinical research, it is important to consider that improvements in the health and well-being of future children can also be achieved by looking at alternative solutions, for instance third party reproduction or adoption. For those limited number of parents for whom PGD is not an option, the choice is not between genome editing and a sick child. The choice is much wider than that. This does not mean that the choice of adopting or relying on third party reproduction comes without a cost, or that prospective parents’ wishes should be neglected. It only means that there are other interests at stake and that there are other strategies than developing new technologies to tackle health needs.

These considerations do not lead to the conclusion that public interest (in the form of a prudent use of resources) should be prioritised over prospective parents’ reproductive autonomy and future offspring’s welfare. On the contrary, the received view, namely the view that considers the interests of these two groups as more morally relevant than those of society, ought to be taken as the default position. But this position should not prevent us from seeking alternatives. Perfecting existing technologies such as PGD, and possibly widening the criteria of access to adoption or third party reproduction, would be a less costly and possibly quicker strategy to grant future children’s welfare while at the same time respecting prospective parents’ wishes. Making existing technologies and practices available via broader state funding schemes would allow their use by larger swaths of the population.

## Conclusions: context matters

In this article, I have analysed the moral case for introducing genome editing as an alternative to PGD. I have presented the reasons in favour and the two main arguments against this possibility, namely safety and germline modifications. After presenting some of the available data on the safety of CRISPR, I have argued that concerns with germline modifications do not represent a compelling argument against the introduction of genome editing into the clinic. I have then turned to arguments in favour of genome editing and concluded that there seems to be a *prima facie* case in favour of starting clinical research with CRISPR. In the last section, I have focused on the moral reasons that are normally taken into account in debates on reproductive technologies, namely the welfare of future children, the reproductive autonomy of the parents and the interests of society. I have showed that a closer look at genome editing in light of these moral reasons seems to generate some additional reasons for caution in accepting genome editing as a new reproductive option. These reasons may entail shifting from funding new resources, such as CRISPR, and advocating for its introduction in the name of values such as reproductive autonomy and the welfare of future children, to focusing on widening the criteria of access to existing options and possibly re-thinking resource allocation and state funding of assisted reproduction. This paper does not attempt to provide decisive arguments in favour of or against the introduction of CRISPR as a new reproductive option. As many have argued, it may be too soon to have a conclusive assessment of this possibility, if only for the dearth of empirical data regarding its safety and feasibility. Rather, this paper offers a basis to begin a discussion on the ethics of introducing genome editing as an alternative to PGD and stresses the need to consider that scientific research does not happen in a vacuum where the soundest theoretical argument wins. Rather, it happens in a context where resources are limited, where genetic parenthood is an important value cherished by many, and where technical solutions are often given preference over other strategies.

## Footnotes

1 Autosomal recessive diseases develop when an individual has two copies of an abnormal gene.

2 Currently, the United Kingdom is the only country that has allowed mitochondrial DNA replacement techniques. Such techniques represent the only existing method for couples where one member is affected by a mitochondrial condition to have genetically related children.

3 The arguments made for CRISPR can be extended also to other future genome editing technologies. Throughout the paper, I use CRISPR and genome editing or gene editing technologies interchangeably.

4 At least when it is about medical conditions, but this is the case in question, so I will not enter into a discussion on so-called cosmetic traits and enhancement.

5 This observation is conditional as it relies on the interpretation of therapy as a practice that can only be defined as such if there is a *person* to be treated (Rulli 2016a).

6 I refer here to the debate on mitochondrial replacement techniques (MRTs) and not strictly on genome editing with CRISPR, as few commentators have dealt specifically with the question of whether genome editing is identity-affecting (for two examples, see: Gyngell et al. 2016; Liao 2017). One of the two techniques for the replacement of faulty mitochondrial DNA, pronuclear transfer (PNT), arguably represents the most similar case to genome editing as, unlike the other technique for the replacement of mitochondrial DNA (maternal spindle transfer—MST), it is applied after the oocytes has been fertilised. The contention, in the case of PNT, is whether this technique is identity-affecting or not, and commentators have presented differing views on this matter (Liao 2017; Palacio-González 2017; Rulli 2016a; Wrigley et al. 2015). While I am aware that PNT and CRISPR are two distinct technologies, PNT arguably represents the most similar case to genome editing as both CRISPR and PNT are applied *after* fertilisation. Hence, other things being equal, arguments concerning whether PNT is identity-affecting or not can also be considered valid in discussions on whether CRISPR is identity-affecting. It must be noted however, that those who explicitly referred to genome editing maintained that it is *not* identity-affecting (Gyngell et al. 2016; Liao 2017). Interestingly, authors who speculatively consider the possibility of using gene therapy on human embryos before the availability of CRISPR are also divided on this issue (Buchanan 1996; McMahan 2006; Sparrow 2008).

7 Despite some challenges, the biological origin (or gametic origin) that a person has is widely considered a necessary condition of what determines the human being that we are. This is well explained by philosopher Derek Parfit’s ‘Origin View’ (or gametic essentialism): “each person has this necessary property: that of having grown from the particular pair of cells from which this person in fact grew” (Parfit 1984, p. 353). In other words, the fact that two gametes came together and generated me is, under this view, considered a necessary condition of my identity: I am the entity that I am by virtue of my gametic origin. Now, this is linked to the discussion of treatment and selection because a technology such as PGD is identity-affecting. In other words, using PGD causes a numerically different person to come into being, namely a different person than the person that would have come into being had PGD not been used. In the case of genome editing, since the intervention takes place *after* fertilisation, the gametic origin of the genetically modified embryo and the gametic origin of the non-genetically modified embryos are identical. In other words, these two embryos are numerically identical. The contention, however, is that gametic origin is only a necessary and not sufficient condition for having a specific identity. Thus, whether genome editing technologies applied to zygotes/embryos cause a different person to come into being or not remains an open question. If they do, then such technologies cannot be considered therapeutic because a different person comes into being due to the use of genome editing. If they do not, they can be considered therapeutic.

8 If genome editing is employed before the 14th day after fertilisation (as it is required by embryos research regulations in the United Kingdom and in many other countries, Hyun et al. 2016), the embryo could still cleave into two (i.e. twinning). In this case, the children that could potentially develop from such embryo will be two. However, twinning occurs spontaneously and it is not influenced by the use of genome editing on the embryo. As a consequence, the use of the technique does not directly affect the numerical identity of the future child/children as it is not the direct causation of the embryo splitting.
